# Efficacy of multi-layered human iPS cell-derived cardiovascular cell sheets in a pacing-induced canine dilated cardiomyopathy model

**DOI:** 10.1186/s13287-026-05207-x

**Published:** 2026-08-19

**Authors:** Yu Shimoyama, Kenji Kakuta, Kiho Araki, Hyoe Komae, Minoru Ono, Jun K. Yamashita

**Affiliations:** 1 iHeart Japan Corporation, Kyoto, Japan; 2 Department of Cardiovascular Surgery, The University of Tokyo Hospital, Tokyo, Japan; 3 Department of Cellular and Tissue Communications, Graduate School of Medicine, The University of Tokyo, Tokyo, Japan

**Keywords:** Regenerative therapy, Human induced pluripotent stem cell, Dilated cardiomyopathy, Heart failure, Biomaterial, Gelatin hydrogel

## Abstract

**Background:**

Dilated cardiomyopathy (DCM) is a progressive, intractable disease that leads to heart failure. Heart transplantation is the only curative treatment; however, access is limited by donor scarcity. Induced pluripotent stem cell (iPSC)-based therapies are attracting attention for DCM, but suitable large-animal models and robust preclinical data have been limited.

**Methods:**

We generated multi-layered cardiovascular cell sheets from human iPSCs by combining cardiomyocytes with endothelial and stromal cells and overcoming stacking limits using interleaved gelatin hydrogel microspheres, yielding a thicker cardiac tissue-like construct (product code: IHJ-301). To enable rigorous testing in non-ischemic heart failure, we established a modified canine rapid-pacing heart failure model that maintains depressed function without mortality by continuing pacing at a slightly reduced rate after induction (Step-Down Pacing Heart Failure model). IHJ-301 was implanted epicardially onto the left ventricular surface via thoracotomy, and cardiac function was assessed by echocardiography and right-heart catheterization.

**Results:**

After 4 weeks of rapid pacing (230 ± 10 bpm), left ventricular ejection fraction (LVEF) was reduced from 77.8 ± 1.1% (pre-pacing) to 44.9 ± 1.9% (*n* = 11) (0 W). Continued pacing at 210 ± 10 bpm for additional 4 weeks resulted in no mortality and maintained depressed function (4 W LVEF 47.3 ± 2.6%). IHJ-301 was implanted at 0 W. At 4 weeks post-implantation (4 W), all animals in the IHJ-301 group (*n* = 5) showed greater functional improvement than sham (*n* = 6). Absolute changes from 0 W to 4 W were: ΔLVEF (%) 9.38 ± 1.47 vs. 1.90 ± 0.34; Δfractional shortening (%) 4.84 ± 0.75 vs. 0.97 ± 0.18; stroke volume (mL/beat) 1.21 ± 1.26 vs. −2.99 ± 0.60; cardiac output (L/min) 0.19 ± 0.19 vs. −0.58 ± 0.12 (all *p* < 0.05).

**Conclusions:**

We established a non-ischemic large-animal heart failure model that sustains depressed function for one month, enabling clear therapeutic readouts. IHJ-301 significantly improved multiple parameters of cardiac function, providing preclinical evidence that IHJ-301 could offer a promising therapeutic option for DCM.

**Supplementary Information:**

The online version contains supplementary material available at 10.1186/s13287-026-05207-x.

## Introduction

Dilated cardiomyopathy (DCM) is characterized by dilation of the ventricles, particularly the left ventricle, accompanied by systolic dysfunction. It is a progressive and intractable disease that often leads to heart failure or fatal arrhythmias, and no curative therapy exists other than heart transplantation [1]. Although viral infection, genetic mutations, and autoimmune mechanisms have been implicated, the etiology remains undetermined in many cases [2–4]. The prevalence of DCM is estimated to be approximately 1 in 2,500 adults worldwide and about 14 per 100,000 adults in Japan [5, 6]. Heart transplantation remains the only curative treatment; however, the number of eligible patients is severely restricted by the limited supply of donor hearts [7]. For example, in Japan, only 50–80 transplantations are performed annually, despite approximately 20,000 patients with DCM [5, 7]. Furthermore, transplanted donor hearts face a significant risk of functional failure beyond ten years after transplantation, caused by progressive diffuse intimal thickening of the coronary arteries [8].

Left ventricular assist devices (LVADs), initially introduced as bridge-to-transplantation therapy, are increasingly used as destination therapy in patients who are not transplant candidates [9, 10]. In Japan, approximately 50 cases per year are currently performed as destination therapy [7, 9]. Although LVADs provide circulatory support, their long-term use carries substantial risks, including cerebrovascular events due to thrombosis, infection, ventricular arrhythmias, right heart failure, and aortic insufficiency [11, 12]. These limitations underscore the urgent need for novel therapeutic strategies for DCM.

Various cell therapies have been explored to date, such as transplantation of mesenchymal stem cells or differentiated cells [13, 14]. Skeletal myoblast sheets, for example, showed promising efficacy in ischemic cardiomyopathy (ICM), but failed to demonstrate statistically significant benefit in a clinical trial for DCM [15]. Recently, induced pluripotent stem cell (iPSC)–based therapies have attracted worldwide attention [16, 17]. Several iPSC-derived cardiac products are already under clinical trials, including cardiomyocyte sheets (jRCT2053190081) and cardiomyocyte spheroids (jRCT2033210163) for ICM. Following them, recently clinical trials for DCM with iPSC-derived cardiomyocyte sheets (jRCT2053230136) and IHJ-301 (jRCT2033240447) have been just launched.

IHJ-301 is an iPSC-derived product developed for clinical application based on the HiCT (human iPSC-derived cardiac tissues) platform. HiCT consists of alternating layers of gelatin hydrogel microspheres and cell sheets composed predominantly of iPSC-derived cardiomyocytes, endothelial cells, and stromal cells [18–20]. This structure enables survival of the graft even in highly stacked layers, preventing ischemic necrosis. Following transplantation into rat hearts, host-derived vasculature infiltrated the graft, ensuring long-term survival. HiCT demonstrated therapeutic efficacy in both rat subacute myocardial infarction [19, 21] and porcine infarction models (unpublished data). IHJ-301 has been developed by adapting this technology into a clinically applicable cell–biomaterial hybrid product. To enable clinical translation, we advanced preclinical studies of IHJ-301; however, for non-ischemic heart failure relevant to DCM, suitable large-animal models were lacking. Rapid ventricular pacing in a dog has often been used as non-ischemic heart failure model [22], but it presents drawbacks that hinder clear assessment of therapeutic efficacy: Sustained high-frequency pacing for approximately 4 weeks induces progressive left ventricular dysfunction and heart failure, yet continuing rapid pacing after model induction often causes premature death [23], whereas discontinuing pacing permits spontaneous recovery of cardiac function [24]. To address these limitations, we developed a modified model in which, after heart failure induction by rapid pacing, pacing was continued at a slightly reduced rate. This approach maintained depressed function without mortality (termed the Step-Down Pacing Heart Failure [SDPHF] model). Using this improved canine model, we conducted a preclinical evaluation of IHJ-301 and demonstrated its therapeutic potential for DCM.

## Methods

### Pacing-induced heart failure model

Beagle dogs (male, 10 kg, KITAYAMA LABES) were used in this study. The study was approved by the Institutional Ethics Committee of Nissei Bilis Co., Ltd. (Osaka, Japan) (Approved: 2023 − 183 and 2024FEB09). All the animals were handled in accordance with the Principles of Laboratory Animal Care (the National Society for Medical Research) and the Guide for the Care and Use of Laboratory Animals (an NIH publication). This study followed the ARRIVE guidelines 2.0.

**Fig. 1:**
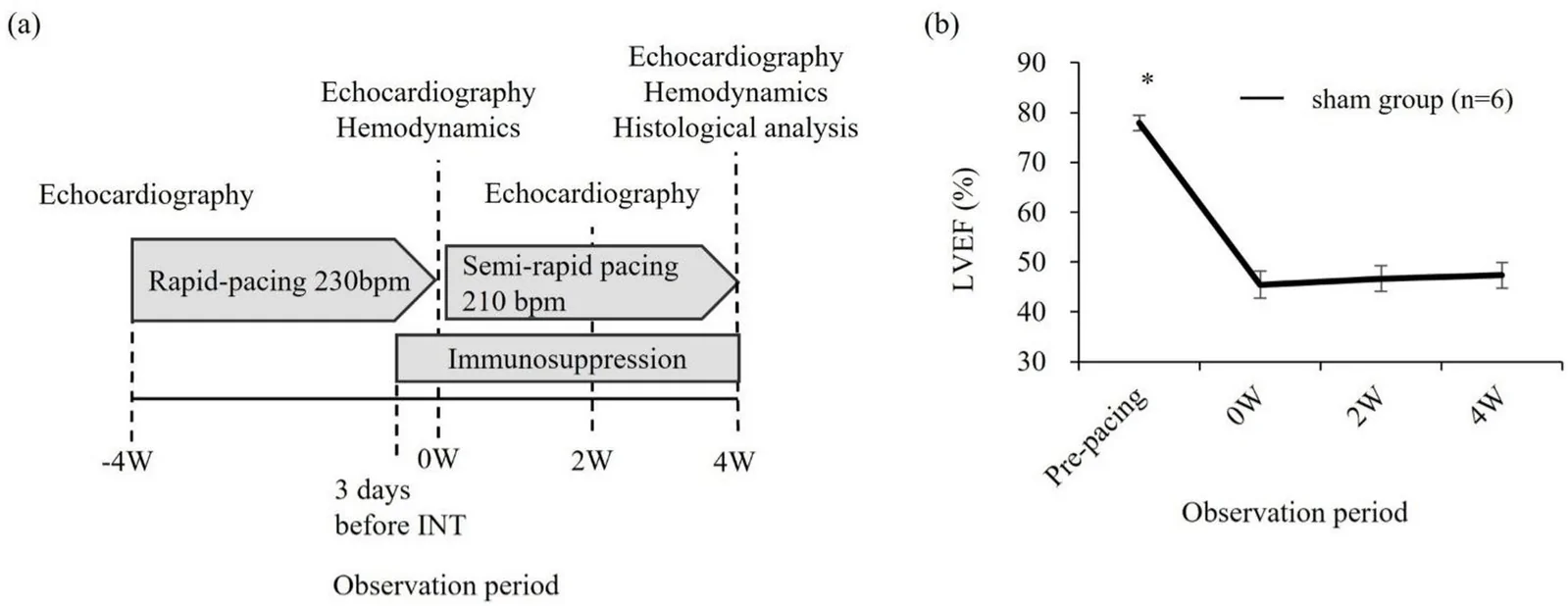
A new pacing-induced heart failure canine model. **a** Experimental design and timeline-4 W: the day of pacemaker implantation (before pacemaker implantation), INT: IHJ-301 implantation or sham operation, 0 W: the day of INT (before INT), 2 W: 2 weeks after INT, 4 W: 4 weeks after INT. **b** LVEF (%) of the model pre-pacing: the day of pacemaker implantation (before pacemaker implantation), 0 W: the day of INT (before IHJ-301 implantation), 2 W: 2 weeks after INT, 4 W: 4 weeks after INT. Asterisk indicated that significant difference (*p* < 0.05) between 0 W and other each observation points with Mann–Whitney U test

A pacemaker, A SIP-501 (STAR MEDICAL, Inc., Tokyo, Japan), was implanted after induction of general anesthesia with 20 mg/kg sodium thiopental, followed by anesthetic maintenance with 1.0–2.0% isoflurane. The pacemaker was implanted subcutaneously in the right jugular region and the tip of a retractable screw-in lead (St Jude Medical, Tokyo, Japan) was placed through the right jugular vein into the right ventricular wall while being checked by a portable fluoroscopic X-ray system, mobile C-arm system (Philips Medical systems, Best, Netherlands). After establishment of the pacing-induced heart failure model, animals were divided into two groups: an IHJ-301 implantation group (IHJ-301 group) and a sham surgical control group (sham group). In both groups, pericardiotomy and application of fibrin glue to the epicardial surface were performed; only the IHJ-301 group received implantation of IHJ-301, whereas the sham group underwent the same surgical procedures without IHJ-301 implantation. The Step-Down Pacing Heart Failure model: Rapid pacing was performed at 230 ± 10 bpm for 4 weeks for model creation, stopped on the day of intervention (INT) for IHJ-301 implantation or sham operation (0 W: the day of INT (before surgery)) and followed by semi-rapid pacing at 210 ± 10 bpm for 4 weeks for model maintenance from 1-day postoperatively (Fig. 1a). Pacemaker operation was confirmed from daily 10-second heart rate (HR) measurements and from the weekly waveform of the lead II ECG. The pacemaker allowed preset pacing rates of 190, 210, 230, or 250 bpm, and correct pacing within ± 10 bpm of the target rate was confirmed by electrocardiography throughout the study period.

For echocardiographic assessment, all animals underwent repeated acclimation and training sessions during the pre-experimental period, allowing rapid and reproducible image acquisition. Echocardiography was performed immediately after pacemaker deactivation once the probe was positioned, minimizing the interval between pacing cessation and measurement. The standardized measurement procedure and consistent HR monitoring were implemented to ensure the consistency of cardiac function assessments across time points.

In accordance with ARRIVE compliance (randomization, blinding, sample size, and exclusions), animals were allocated to IHJ-301 and sham groups 27–28 days after pacing using a deterministic minimization procedure to balance baseline LVEF (%) and LVIDd (mm). Surgeons, outcome measurers, outcome assessors, animal caretakers, and statisticians were assigned independent roles, and outcome assessment and statistical analysis were performed using coded group identifiers. Sample size (*n* = 6 per group) was determined a priori based on ΔLVEF as the primary endpoint. One animal in the IHJ-301 group subsequently reached a humane endpoint and was excluded from the analysis, resulting in final analyzed group sizes of 6 in sham and 5 in IHJ-301; details are provided in Results Sect.  2. Inclusion criteria were prespecified as animals that demonstrated normal weight gain and no abnormalities in general condition during the quarantine and acclimation period and exhibited a reduction in LVEF (%) to 30–55% after rapid pacing, whereas exclusion criteria included animals that failed to meet these conditions.

### Cell culture, cell differentiation, sheet production and implantation

Human iPSCs (QHJI0104s) were provided by CiRA, Kyoto University [25]. iHeart Japan Corporation established a cell bank from QHJI01s04 cells under GMP-compliant conditions (Takara Bio Inc., Kusatsu, Japan). Cells from the bank were then used for all experiments.

IHJ-301 was provided by iHeart Japan Corporation (Kyoto, Japan), and all IHJ-301 products utilized in this study met the manufacturer’s predefined release criteria. Thirty-five mm-diameter cell sheets used in the IHJ-301 were generated based on our previous study [26]. All other biological and structural characteristics of IHJ-301 were comparable to HiCT using clinical grade human iPSCs [21].

### Immunosuppression

Tacrolimus (TAC), mycophenolate mofetil, and prednisolone were used as immunosuppressants at doses of 1 mg/kg/day, 20 mg/kg/day and 1 mg/kg/day, respectively, based on previously reported protocols for canine experimental transplantation models [27, 28]. Immunosuppressants were administered orally from 3 days before to 27 days after INT.

### Blood chemistry tests

Blood samples were collected from the flexor cutaneous vein at 0 (0 W), 1, 2 (2 W: 2 weeks after the INT), 3 and 4 (4 W: 4 weeks after the INT) weeks after the operation into sodium heparin mixed blood collection tubes without anesthesia and the plasma was obtained via centrifugation at 3,000 rpm for 10 min, at 4℃. Blood sampling at 0 W was performed in the morning on the day of examination, whereas samples at 4 W were collected immediately prior to euthanasia.

The tests items consisted of transaminases [ASAT (GOT), ALAT (GPT)], alkaline phosphatase (ALP), lactate dehydrogenase (LDH), leucine aminopeptidase activity (LAP), cholinesterase activity (ChE), γ-glutamyl transpeptidase (γ-GTP), total bilirubin (TB), total protein (TP), albumin (Alb), total cholesterol (TC), triglycerides (TG), urea nitrogen (BUN), uric acid (UA) and creatinine (Cre). These parameters were measured using a Hitachi automated analyzer (Hitachi high-tech Ltd., Tokyo, Japan). The plasma concentrations of TAC were measured with an FK506 ELISA kit (Abnova, Taipei City, Taiwan).

### Observation of general conditions and body weight

The general condition was observed daily for posture, activity, gum color, presence of ascites accumulation, stools, and food intake. Body weight was measured using an electronic balance on the day of pacemaker implantation and every week thereafter.

### Measurement of cardiac function with echocardiography

Echocardiographic examination was performed before pacemaker implantation (pre-pacing), on the day of the INT (0 W), and 2 W and 4 W. The pacemaker was temporarily stopped during the echocardiographic examination, which was performed without anesthesia. Pacemaker deactivation was confirmed by lead II ECG. Echocardiography was performed via a general-purpose ultrasound imaging system (Vivid S6, GE Medical Systems) with a Kutter probe (11 MHz) in the right chest in M-mode. The measurements included left ventricular end-diastolic diameter (LVIDd), left ventricular end-systolic diameter (LVIDs), left ventricular ejection fraction (LVEF) and fraction shortening (FS). The pacemaker was restarted at semi-rapid pacing after the end of the echocardiographic examination. The pacing rate after pacemaker restart was confirmed by lead II ECG.

### Measurement of cardiovascular and hemodynamic parameters

Cardiovascular and hemodynamic parameters were assessed at baseline (0 W) and at 4 W. All monitoring devices and pressure transducers were calibrated before each experiment in accordance with the manufacturers’ instructions and the standard operating procedures of Nissei Bilis to ensure measurement accuracy and reproducibility. After induction of general anesthesia, a Swan-Ganz thermodilution catheter (5 Fr; 132F5, Edwards Lifesciences Inc., Tokyo, Japan) was inserted via the femoral vein and advanced into the pulmonary artery under fluoroscopic guidance using a mobile C-arm system. The catheter was connected to a blood pressure monitoring kit (DX-300, Nihon Kohden Co., Ltd., Tokyo, Japan) via a measuring amplifier (coupler amplifier PP-101 H/AP-100 H, Nihon Kohden Co., Ltd.), allowing continuous measurement of right atrial pressure (RAP), pulmonary artery pressure (PAP), and pulmonary capillary wedge pressure (PCWP). Cardiac output (CO) was measured using a thermodilution cardiac output monitor (MTC-6210, Nihon Kohden Co., Ltd.). A bolus of 5 mL ice-cold 5% glucose solution was injected via the thermodilution catheter using a 5 mL syringe following completion of PCWP measurement. Each CO measurement consisted of a single 5-second recording after bolus injection.

Hemodynamic measurements were performed in a fixed sequence (RAP, PAP, PCWP, and CO) at each time point to minimize procedural variability. For each parameter, two consecutive measurements were obtained under stable hemodynamic conditions, and the mean value was used for subsequent analysis. Heart rate (HR) was derived from electrocardiographic recordings obtained in lead II using a polygraph system (RMT-1000, Nihon Kohden Co., Ltd.) with a measuring amplifier (PB-101 H/ AB-100 H, Nihon Kohden Co., Ltd.). ECG waveforms were digitally recorded and analyzed using LabChart Pro software (AD Instruments, Nagoya, Japan). Hemodynamic recordings were analyzed by investigators blinded to group allocation.

### Histological analysis

Upon completion of cardiovascular and hemodynamic tests at 4 W, the animals were euthanized under deep anesthesia with isoflurane via exsanguination. The heart and lungs were immediately removed, and the blood was removed with saline solution. The heart (left ventricle) was fixed by immersion in a 10% neutral buffered formalin solution. The left ventricle was divided into eight sections in the short-axis direction near the papillary muscle, and a paraffin block was prepared with the basal side of each section as the embedding plane. After the paraffin blocks were prepared, they were thinly sectioned and subjected to hematoxylin-eosin (HE) staining and Masson trichrome (MT) staining.

### Statistics

All values are presented as means ± standard errors. The primary endpoint of this study was ΔLVEF (%) from baseline (0 W) to 4 W. Between-group differences in the primary endpoint were analyzed using the Mann–Whitney U test as the primary statistical analysis. LVEF (%), FS (%), LVIDd (mm), LVIDs (mm), SV, and CO were prespecified as secondary endpoints. For these secondary endpoints, both observed values at each time point and changes from 0 W (ΔFS, ΔSV, and ΔCO) were evaluated. Between-group comparisons for all secondary endpoints and their changes from baseline were conducted using the Mann–Whitney U test as the primary nonparametric analysis. For the measured values of LVEF, FS, LVIDd, and LVIDs, baseline-adjusted comparisons were further assessed using rank-based ANCOVA as supportive analyses. In addition, longitudinal changes in the primary endpoint (ΔLVEF), and secondary endpoints were explored using mixed-effects models including time (0 W and 4 W), treatment group (IHJ-301 vs. sham), and their interaction as fixed effects, with a random intercept for each animal to account for within-subject correlation. Effect estimates are reported as adjusted mean differences with corresponding 95% confidence intervals. All statistical analyses were performed using R Commander (version 2.7-1) [29], and a two-sided *P* value < 0.05 was considered statistically significant.

## Results

### No serious blood chemistry events with IHJ-301

The plasma TAC concentrations (ng/mL) on 0 W and 4 W in the IHJ-301 group (0.90 ± 0.22 and 0.48 ± 0.13, respectively) and the sham group (0.93 ± 0.41 and 0.47 ± 0.07, respectively) were not significantly different between the two groups.

The results of blood biochemistry tests showed significantly higher LAP in the IHJ-301 group than in the sham group at 4 W, though those remained within normal limits. The other values of liver function and renal function test were not significantly different between the two groups at 4 W (Table 1).

**Table 1:** Plasma tacrolimus concentrations and blood biochemistries

	Sham group (*n* = 6)		IHJ-301 group (*n* = 5)	
	0 W	4 W	0 W	4 W
Tac (ng/mL)	0.93 ± 0.41	0.47 ± 0.07	0.90 ± 0.22	0.48 ± 0.13
ASAT(GOT) (IU/L)	24 ± 1	20 ± 1	26 ± 4	18 ± 1
ALAT(GPT) (IU/L)	48 ± 5	23 ± 3	66 ± 15	27 ± 4
ALP (IU/L)	103 ± 6	147 ± 41	104 ± 20	82 ± 17
LDH (IU/L)	47 ± 6	40 ± 7	41 ± 5	35 ± 4
LAP (IU/L)	29 ± 3	17 ± 1	34 ± 3	21 ± 1*
ChE (IU/L)	12 ± 1	7 ± 1	11 ± 1	6 ± 1
γ-GTP (IU/L)	5 ± 1	5 ± 1	5 ± 0	5 ± 1
TB (mg/dL)	0.04 ± 0.01	0.03 ± 0.01	0.03 ± 0.01	0.02 ± 0.01
TP (g/dL)	6.4 ± 0.1	6.2 ± 0.1	6.3 ± 0.2	6.0 ± 0.1
Alb (g/dL)	3.4 ± 0.1	3.4 ± 0.1	3.3 ± 0.1	3.4 ± 0.1
A/G	1.14 ± 0.05	1.22 ± 0.07	1.11 ± 0.05	1.28 ± 0.06
TC (mg/dL)	122 ± 7	130 ± 11	156 ± 10	150 ± 20
TG (mg/dL)	65 ± 12	60 ± 8	69 ± 10	62 ± 11
BUN (mg/dL)	25.3 ± 2.4	17.2 ± 1.9	21.3 ± 1.6	18.6 ± 1.2
UA (mg/dL)	0.26 ± 0.02	0.24 ± 0.04	0.28 ± 0.02	0.16 ± 0.02
Cre (mg/dL)	0.59 ± 0.05	0.53 ± 0.03	0.58 ± 0.03	0.48 ± 0.02

Sham group: surgical control without IHJ-301 implantation, IHJ-301 group: surgical implantation of IHJ‑301, 0 W: the day of INT (before surgery), 4 W: 4 weeks after INT, Tac: plasma concentration of tacrolimus, ASAT: aspartate transaminase, ALAT: alanine transaminase, ALP: alkaline phosphatase, LDH: lactate dehydrogenase, LAP: leucine Aminopeptidase, ChE: cholinesterase, γ-GTP: gamma-glutamyltransferase, TB: total bilirubin, TP: total protein, Alb: albumin, A/G: albumin/globulin, TC: total cholesterol, TG: triglyceride, BUN: blood urea nitrogen, UA: uric acid, Cre: creatinine

Asterisk indicated that significant difference between sham and IHJ-301 groups

### No serious general conditions with IHJ-301

Sporadic soft feces or diarrhea was observed after the administration of immunosuppressants in both groups. Body weight (kg) was measured on pre-pacing, 0 W, 2 W and 4 W, respectively in the IHJ-301 group (10.16 ± 0.11, 9.91 ± 0.13, 9.63 ± 0.12 and 9.60 ± 0.13, respectively) and the sham group (10.49 ± 0.10, 10.18 ± 0.20, 9.64 ± 0.20 and 9.62 ± 0.32, respectively). Significant weight loss was observed in both groups at 2 W and 4 W compared with pre-pacing (Table 2). Although no animals were excluded at the time of allocation, one animal in the IHJ-301 group reached a humane endpoint and was subsequently excluded from the analysis. This individual exhibited progressive emaciation due to anorexia; however, a post-mortem necropsy identified intussusception. This condition was judged to be a complication associated with the immunosuppressive regimen and was determined to be independent of the IHJ-301 treatment.

**Table 2:** Body weight (kg)

sham group (*n* = 6)	Pre-pacing	0 W	2 W	4 W
	10.49 ± 0.10	10.18 ± 0.20	9.64 ± 0.20*	9.62 ± 0.32*
IHJ-301 group (*n* = 5)	10.16 ± 0.11	9.91 ± 0.13	9.63 ± 0.12*	9.60 ± 0.13*

sham group: surgical control without IHJ-301 implantation, IHJ-301 group: surgical implantation of IHJ‑301, pre-pacing: the day of pacemaker implantation (before implantation), 0 W: the day of INT (before implantation), 2 W: 2 weeks after INT, 4 W: 4 weeks after INT

Asterisks indicate significant differences between pre-pacing and each observation point within each group

### A model with sustained heart failure without mortality

The LVEF (%) was measured with echocardiography at the time of pre-pacing, 0 W, 2 W and 4 W. Following pacemaker implantation and rapid pacing, the sham group showed a marked reduction in LVEF (%) at 0 W. Importantly, the impaired LVEF (%) was sustained during an additional 4 weeks of semi-rapid pacing (210 bpm), without any animal death (Fig. 1b). These results demonstrate that our new protocol, termed the Step-Down Pacing Heart Failure (SDPHF) model (Fig. 1a), provides a large-animal model that enables long-term evaluation of non-ischemic heart failure without animal mortality.

### Improvements in cardiac function with IHJ-301 treatment

**Fig. 2:**
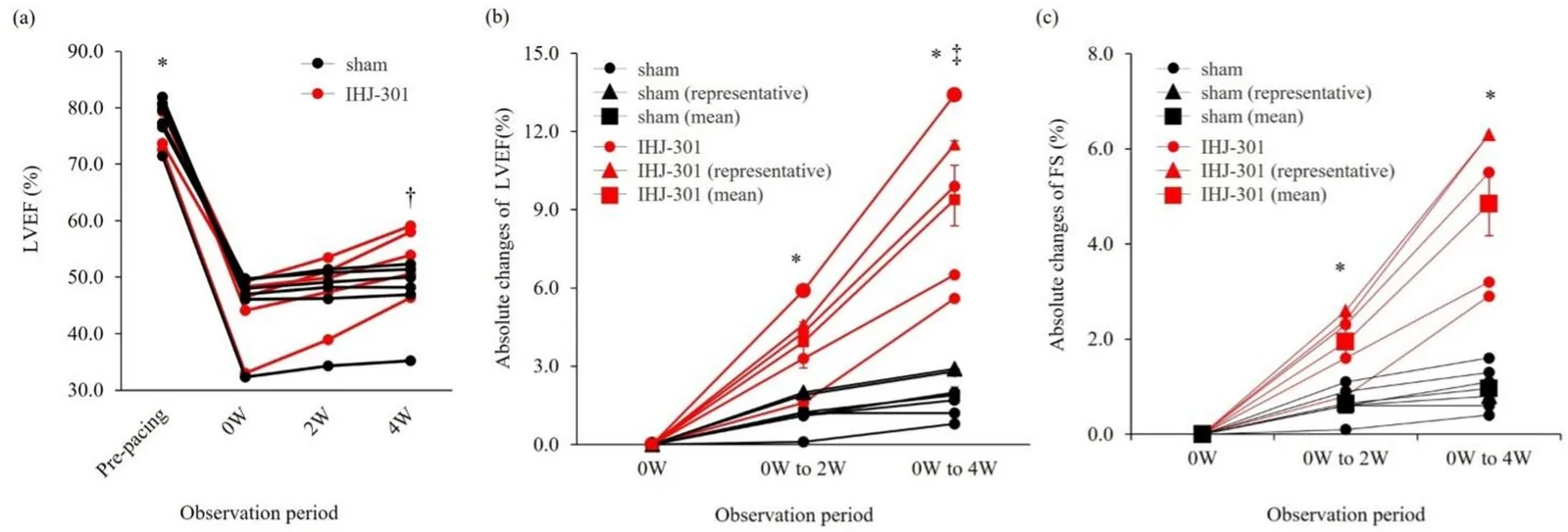
Echocardiography findings, **a** Changes of LVEF (%) pre-pacing: the day of pacemaker implantation (before pacemaker implantation), 0 W: the day of INT (before IHJ-301 implantation), 2 W: 2 weeks after INT, 4 W: 4 weeks after INT. Asterisk and single dagger indicate statistically significant differences (*p* < 0.05) compared with 0 W, as determined by the Mann–Whitney U test and rank-based ANCOVA, respectively, within the IHJ-301 and sham groups (*n* = 5 and 6, respectively). **b** Absolute changes of LVEF (%) after INT 0 W: the day of INT (before IHJ-301 implantation), 0 W to 2 W: absolute changes of LVEF (%) between 0 W and 2 W, 0 W to 4 W: absolute changes of LVEF (%) between 0 W and 4 W. sham (representative): represented sham operated animal in Fig. 3. sham (mean): average of LVEF (%) in sham group (*n* = 6). IHJ-301 (representative): represented IHJ-301 implanted animal in Fig. 3. IHJ-301 (mean): average of LVEF (%) in IHJ-301 group (*n* = 5). Asterisk and double dagger indicate statistically significant differences (*p* < 0.05) compared with 0 W, as determined by the Mann–Whitney U test and a linear mixed-effects model, respectively, within the IHJ-301 and sham groups (*n* = 5 and 6, respectively). (c) Absolute changes of FS (%) with INT. 0 W: the day of INT (before IHJ-301 implantation), 0 W to 2 W: absolute changes of FS (%) between 0 W and 2 W, 0 W to 4 W: absolute changes of FS (%) between 0 W and 4 W. sham (representative): represented sham operated animal in Fig. 3. sham (mean): average of FS (%) in sham group (*n* = 6). IHJ-301 (representative): represented IHJ-301 implanted animal in Fig. 3. IHJ-301 (mean): average of FS (%) in IHJ-301 group (*n* = 5). Asterisk indicated that significant difference (*p* < 0.05) between IHJ-301 and sham groups at each observation point

Using SDPHF model, we estimated the effects of IHJ-301 treatment on non-ischemic heart failure. The LVEF (%) was measured with echocardiography at the time of pre-pacing, 0 W, 2 W and 4 W in the IHJ-301 group (77.6 ± 1.8, 43.2 ± 2.6, 48.2 ± 2.5 and 53.6 ± 2.4, respectively) and the sham group (78.0 ± 1.5, 45.4 ± 2.7, 46.7 ± 2.6 and 47.3 ± 2.6, respectively) (Table 3) Similar to the sham group, the IHJ-301 group showed a marked reduction in LVEF (%) at 0 W. In contrast, the IHJ-301 group exhibited a clear trend toward recovery of LVEF (%) at 4 W compared with the sham group (Red lines, Fig. 2a). Whereas simple comparison of LVEF (%) between the sham and IHJ-301 groups at 4 W did not reach statistical significance (*p* = 0.056), between-group comparisons using rank-based ANCOVA with baseline adjustment demonstrated a significant improvement in LVEF (%) favoring the IHJ-301 group at 4 W (adjusted rank difference 3.81, 95% CI 1.61–6.00; *p* = 0.0039). The FS (%) was measured with echocardiography at the time of pre-pacing, 0 W, 2 W and 4 W in the IHJ-301 group (38.6 ± 1.6, 17.3 ± 1.2, 19.7 ± 1.3 and 22.6 ± 1.3, respectively) and the sham group (39.7 ± 1.4, 18.4 ± 1.3, 19.0 ± 1.5 and 19.4 ± 1.2, respectively) (Table 3). In the sham group, the depressed FS (%) was sustained throughout the study period, similar to LVEF (%). Between-group comparisons using rank-based ANCOVA with baseline adjustment demonstrated a significant improvement in FS (%) favoring the IHJ-301 group at 4 W (adjusted rank difference 3.81, 95% CI 1.61–6.00; *p* = 0.0039). A trend favoring the IHJ-301 group was observed in both LVEF (%) and FS (%) at 2 W.

The absolute changes in LVEF (%) (ΔLVEF) and FS (%) (ΔFS) from 0 W showed clear differences between the IHJ-301 and sham groups (Fig. 2b and c). In the IHJ-301 group, significant increases in ΔLVEF and ΔFS were observed from 2 W (*p* = 0.022 and 0.021, respectively), and these improvements became more pronounced at 4 W (*p* = 0.004 and 0.008, respectively). Notably, all five animals in the IHJ-301 group demonstrated recovery values exceeding those of the sham group. ΔLVEF from baseline were further analyzed using a linear mixed-effects model with time (0 W, 2 W, and 4 W), treatment group (IHJ-301 group vs. sham group), and their interaction as fixed effects, and subject as a random effect, with baseline (0 W) defined as the reference time point.

**Fig. 3:**
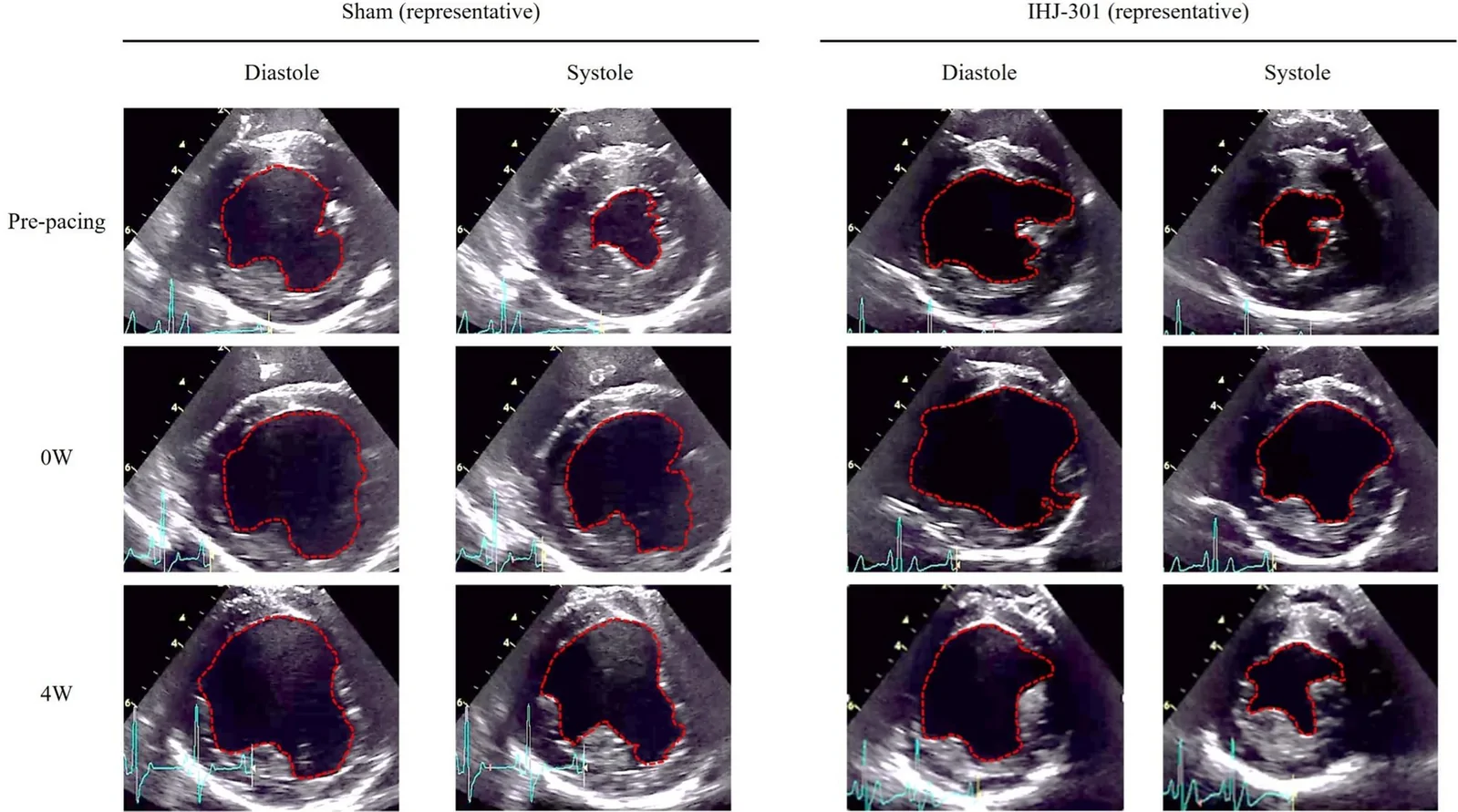
Improvement of contractility with IHJ-301. Representative diastolic and systolic images from echocardiography. pre-pacing: the day of pacemaker implantation (before pacemaker implantation), 0 W: the day of INT (before INT), 4 W: 4 weeks after INT. Images of sham and IHJ-301 operated animals indicated black and red triangles in Fig. 2b, respectively. Red dotted lines indicated left ventricular endocardial wall

At 2 W, the increase in LVEF(%) from baseline (0 W) in the IHJ-301 group was significantly greater than that in the sham group (adjusted mean difference 2.71% points, 95% CI 1.08–4.33; *p* = 0.004). At 4 W, this between-group difference was further increased, with a significantly greater improvement in the IHJ-301 group compared with the sham group (adjusted mean difference 7.48% points, 95% CI 4.35–10.61; *p* < 0.001). Representative echocardiographic images are shown in Fig. 3 and Video S1-S6 (corresponding to the samples marked with triangles in Fig. 2b and c, respectively). While the sham group exhibited no recovery of the reduced contractility, the IHJ-301 group showed consistent improvement in left ventricular contractility. (Echocardiographic images of all other samples are presented in Figure S1).

**Table 3:** Echocardiography

	Sham group (*n*=6)				IHJ-301 group (*n*=5)			
	pre-pacing	0 W	2 W	4 W	pre-pacing	0 W	2 W	4 W
LVEF (%)	78.0±1.5	45.4±2.7	46.7±2.6	47.3±2.6	77.6±1.8	43.2±2.6	48.2±2.5	53.6±2.4^†^
FS (%)	39.7±1.4	18.4±1.3	19.0±1.5	19.4±1.2	38.6±1.6	17.3±1.2	19.7±1.3	22.6±1.3^†^
LVIDd (mm)	32.1±0.7	38.8±0.9	36.9±1.2	35.5±1.9	33.4±0.9	38.4±1.3	38.3±1.7	36.6±1.8
LVIDs (mm)	19.4±0.7	31.7±1.1	29.9±1.2	28.7±2.0	20.5±1.0	31.8±1.5	30.8±1.7	28.3±1.7

sham group: surgical control without IHJ-301 implantation, IHJ-301 group: surgical implantation of IHJ‑301, pre-pacing: the day of pacemaker implantation (before implantation), 0 W: the day of INT (before implantation), 2 W: 2 weeks after INT, 4 W: 4 weeks after INT, LVEF (%): % left ventricular ejection fraction (%), FS (%): % fractional shortening, LVIDd (mm): diastolic left ventricular internal dimension, LVIDs (mm): systolic left ventricular internal dimension

Single dagger indicate statistically significant differences (*p* < 0.05) compared with 0 W, as determined by the rank-based ANCOVA within the IHJ301 and sham groups (*n* = 5 and 6, respectively)

### Efficacies for cardiovascular and hemodynamic functions

**Fig. 4:**
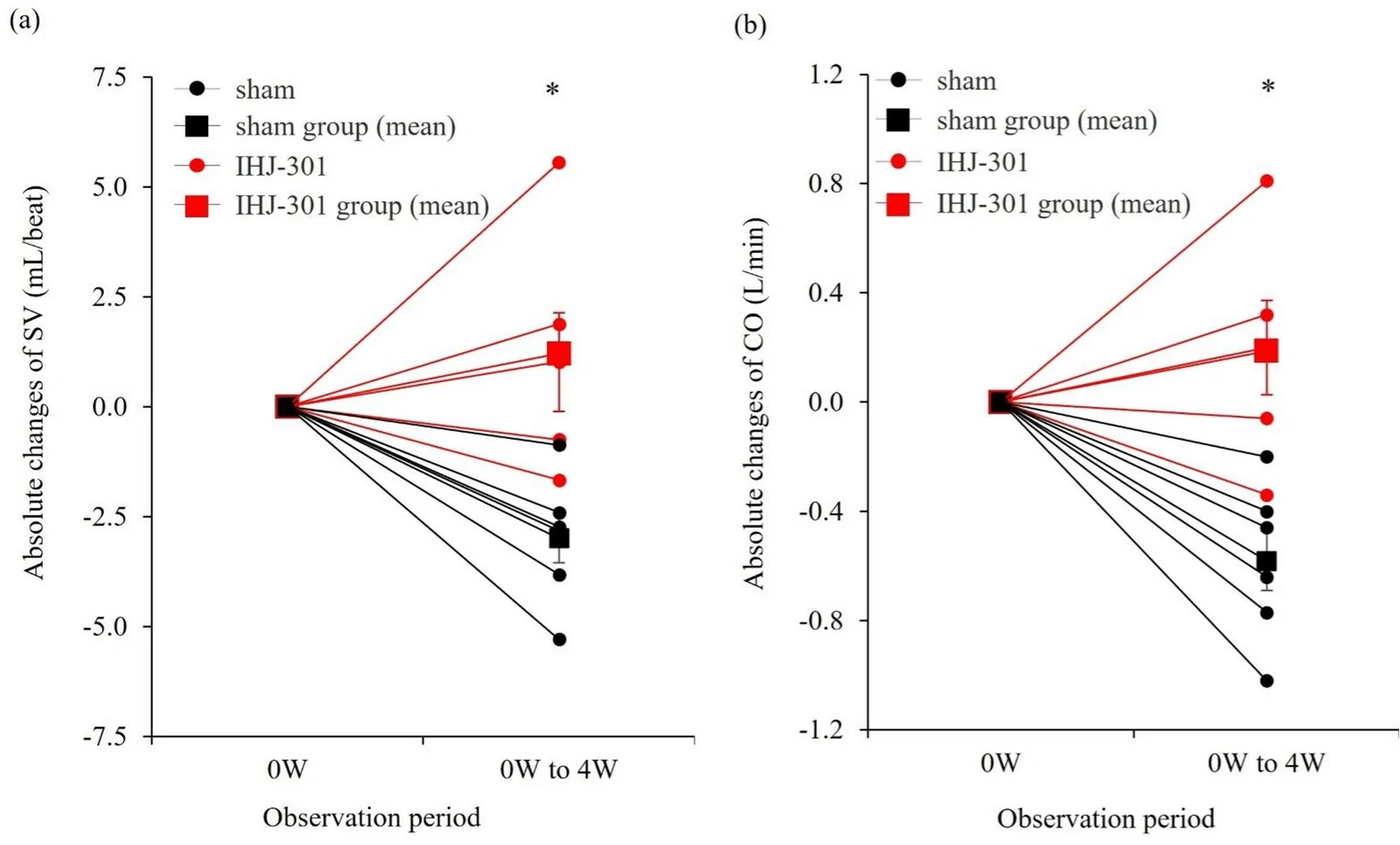
Cardiovascular and hemodynamic findings with right heart catheterization. **a** Changes of SV (mL/beat) with INT. 0 W: the day of INT (before implantation), 4 W: 4 weeks after INT. sham group (mean): average of SV in sham group (*n* = 6). IHJ-301 group (mean): average of SV in IHJ-301 group (*n* = 5). Asterisk indicated that significant difference (*p* < 0.05) between IHJ-301 and sham groups. **b** Changes of CO (L/min) with INT. 0 W: the day of INT (before implantation), 4 W: 4 weeks after INT. sham (mean): average of CO in sham group (*n* = 6). IHJ-301 (mean): average of CO in IHJ-301 group (*n* = 5). Asterisk indicated that significant difference (*p* < 0.05) between IHJ-301 and sham groups

Hemodynamic assessment using a Swan-Ganz catheter also demonstrated functional improvement by IHJ-301 (Fig. 4a and b; Table 4). At 0 W, stroke volume (SV, mL/beat) was significantly lower in the IHJ-301 group (SV 8.33 ± 0.44, CO 1.11 ± 0.09) than in the sham group (SV 10.79 ± 0.83, CO 1.57 ± 0.19), indicating that heart failure was more severe in the IHJ-301 group at baseline (0 W). By 4 W, however, the sham group showed further deterioration (SV 7.80 ± 0.36, CO 0.99 ± 0.10), whereas the IHJ-301 group exhibited improvement in both parameters (SV 9.58 ± 1.04, CO 1.33 ± 0.15). From 0 W to 4 W, all sham animals showed decreases in SV and CO. In contrast, despite some inter-animal variability, three of five IHJ-301-treated animals showed increases in these parameters, leading to a significant difference in overall recovery between sham and IHJ-301 groups by the Mann–Whitney U test (*p* = 0.009) (Fig. 4a and b). In addition, changes of SV and CO from baseline (0 W) were analyzed using a linear mixed-effects model including time (0 W and 4 W), treatment group (IHJ-301 vs. sham), and their interaction as fixed effects, with a random intercept for each subject. At 4 W, the IHJ-301 group showed a significantly greater increase from baseline (0 W) compared with the sham group. The adjusted mean difference (IHJ-301 − sham) of CO and SV were 0.77 units (95% CI 0.38–1.15; *p* = 0.0001) and 4.2 units (95% CI 1.0–7.4; *p* = 0.01), respectively (Table 4). The values of RAP, PAP, PCWP and HR had no significant difference between the two groups at each observation point.

**Table 4:** Hemodynamics

	Sham group (*n* = 6)		IHJ-301 group (*n* = 5)	
	0 W	4 W	0 W	4 W
RAP (mmHg)	4.6 ± 0.8	2.9 ± 0.3	6.4 ± 1.6	2.6 ± 0.3
PAP (mmHg)	14.9 ± 1.9	9.0 ± 1.4	14.1 ± 1.4	11.2 ± 0.7
PCWP (mmHg)	7.0 ± 0.9	3.8 ± 0.3	6.6 ± 0.8	4.4 ± 0.4
HR (beats/min)	144 ± 8	126 ± 7	134 ± 10	139 ± 7
SV (mL/beat)	10.79 ± 0.83	7.80 ± 0.36	8.33 ± 0.44*	9.58 ± 1.04^‡^
CO (L/min)	1.57 ± 0.19	0.99 ± 0.10	1.11 ± 0.09	1.33 ± 0.15^‡^

sham group: surgical control without IHJ-301 implantation, IHJ-301 group: surgical implantation of IHJ‑301, 0 W: the day of INT (before implantation), 4 W: 4 weeks after INT, RAP: Right atrium pressure, PAP: Pulmonary artery pressure, PCWP: Pulmonary capillary wedge pressure, HR: heart rate, SV: Stroke volume, CO: Cardiac output

Asterisk and double dagger indicate statistically significant differences (*p* < 0.05) compared with 0 W, as determined by the Mann–Whitney U test and a linear mixed-effects model respectively, within the IHJ-301 and sham groups (*n* = 5 and 6, respectively)

### Long-term survival of IHJ-301

Histological analysis confirmed engraftment of IHJ-301 in all implanted animals (5/5) on 4 W. As shown in representative low magnification views of MT staining, fibrotic changes within the cardiac tissue were minimal and comparable in the two groups (sham 1.0 ± 0.2%, IHJ-301 0.6 ± 0.1%) (Table S1 and Figure S2 a and b). Although limited fibrosis and fibrin deposition were observed only adjacent to the graft, likely reflecting the implantation procedure and the fibrin gel used for graft fixation, no apparent inflammatory response was observed in either the sham or IHJ-301 group (Table S2 and Figure S2b and c). A higher-magnification view of H&E staining showed a thickened epicardial region containing IHJ-301, together with residual gelatin hydrogel microspheres. Numerous small blood vessels were observed within and around this region, and the thickness of the epicardial graft-containing area reached approximately 400–600 μm (Figure S2c).

## Discussion

In this study, we developed an improved canine rapid pacing model of non-ischemic heart failure, in which cardiac dysfunction was maintained for one month after induction with no mortality (SDPHF model). Using this DCM-like model, we demonstrated that IHJ-301, a hybrid product composed of human iPSC-derived cardiovascular cells and biomaterials, promoted functional recovery. These findings provide important preclinical evidence supporting IHJ-301 as a promising therapeutic option for DCM.

Large-animal models that faithfully recapitulate the pathophysiology of DCM remain limited. Although genetically modified porcine models have been reported, they require genetic engineering and are therefore not widely feasible [30]. Canine pacing-induced models have also been used, but, as noted in the Introduction, they have several limitations [23, 24, 31]. In the present study, we addressed these limitations by introducing a simple and broadly applicable modification to the canine pacing model, in which the pacing frequency was moderately reduced after heart failure induction. As shown in Fig. 2a, 4 weeks of high-frequency pacing reduced LVEF from normal levels (> 70%) to approximately 50% or lower in all animals, representing a clear heart-failure phenotype. Thereafter, during an additional 4 weeks of step-down pacing, little spontaneous recovery was observed. Notably, even in the most severely affected animal, in which LVEF declined to 32.3%, depressed cardiac function was maintained without mortality. Thus, this method successfully established heart failure in all six animals while avoiding increased mortality, indicating a high success rate and practical utility. Taken together, this approach may provide a new, simple, and stable experimental platform for large-animal studies of non-ischemic heart failure.

Using this model, we evaluated the therapeutic effects of IHJ-301. The primary endpoint, ΔLVEF from 0 W to 4 W, showed a significant improvement (+ 9.38%), indicating therapeutic efficacy in this non-ischemic heart failure model. Among the secondary endpoints, significant improvements were also observed in ΔFS (+ 4.84%), SV (8.33 mL/beat at 0 W and 9.58 mL/beat at 4 W), and CO (1.11 L/min at 0 W and 1.33 L/min at 4 W). Direct cross-sectional comparison of absolute LVEF values between the sham and IHJ-301 groups at 4 W did not reach statistical significance, likely owing in part to the relatively large baseline dispersion, including animals with markedly depressed cardiac function at 0 W. Consistent with this interpretation, baseline-adjusted analysis (ANCOVA) demonstrated significant improvement in both LVEF and FS. As shown in Fig. 2a, there were sham-treated and IHJ-301-treated animals with similarly severe reductions in LVEF at 0 W (approximately 30%); even in such severely affected cases, IHJ-301 treatment was associated with a clear trend toward functional improvement compared with sham treatment. The longitudinal analyses of individual trajectories were also supportive for the functional improvement in the IHJ-301 group. Taking together, the consistent improvement observed across both echocardiographic and catheter-based hemodynamic assessments supports the therapeutic potential of IHJ-301 in this non-ischemic heart failure model, one experimental representation of DCM.

Regarding immunosuppression, a tacrolimus-based regimen commonly used in canine experimental transplantation models was applied. In the present study, dosing was determined according to these published protocols, and tacrolimus blood concentrations were not quantitatively evaluated [27, 28, 32]. Because appropriate target blood concentrations of tacrolimus in dogs have not been clearly established, a detailed pharmacokinetic assessment was challenging. Notably, all animals that received tacrolimus exhibited body weight loss after the initiation of immunosuppressive treatment, a finding consistent with known adverse effects of tacrolimus. Optimization of the immunosuppressive regimen— including dosing strategy, blood concentration monitoring, and mitigation of adverse effects—will be required in future studies.

With respect to the mechanism of action, it remains unclear whether IHJ-301 exerts its effect through physical contractile support by synchronized graft cardiomyocytes. Instead, paracrine mechanisms are likely to contribute substantially, as suggested by previous studies of cardiac sheet-like structure transplantation [18, 33–35]. Recently, three major candidates mediating such paracrine effects have been proposed: cytokines, extracellular vesicles (EVs), and mitochondria transfer from implanted cells [36–39]. In the present study, no ischemia or overt cardiac fibrosis was observed in failing hearts. These findings may argue against a major contribution of pathways associated with angiogenesis, anti-fibrotic effects, or inhibition of apoptosis, which are often discussed in the context of cytokine or EV-mediated mechanisms. Recent attention has focused on horizontal mitochondrial transfer as a potential mechanism of intercellular communication. Cardiomyocytes are rich in mitochondria, and mitochondria have been reported to be released into culture supernatants from cardiomyocytes [40]. Given that mitochondria are critical organelles for energy production and that mitochondrial transplantation has been shown to improve cardiac function [41, 42], horizontal transfer of mitochondria may represent one possible mechanism contributing to the therapeutic effect of IHJ-301. In addition, other mechanisms—including effects on microvascular function, myocardial energetics, or inflammatory modulation—may also have contributed to the observed functional improvement. Further studies will be required to distinguish among these possibilities and to determine the relative contribution of each mechanism.

Another key feature of IHJ-301 (based on HiCT technology) is its long-term engraftment. Unlike conventional non-layered cardiomyocyte sheets, which typically disappear within 1–2 months after transplantation, HiCT constructs are supported by host-derived neovascular networks, allowing long-term survival (at least three months) in rodent models [19, 21]. A substantial IHJ-301 mass still persisted at least one month post-transplantation in the present study (Figure S2c), suggesting the potential for sustained paracrine activity. Accordingly, IHJ-301 may function as an in situ paracrine organoid adjacent to the diseased myocardium, continuously supplying cytokines, EVs, mitochondria, and other factors that may augment host cardiac function. Although this proposed mechanism remains speculative, it may contribute to the therapeutic benefits observed in this study. If supported by further mechanistic studies, such a property could represent a distinctive therapeutic advantage of IHJ-301 for DCM. Collectively, our results provide preclinical support for ongoing clinical trials of IHJ-301 in patients with DCM (jRCT 2033240447).

## Conclusion

We established a large-animal non-ischemic heart failure model that stably maintains depressed function without mortality. In this model, IHJ-301 improved multiple indices of cardiac function, supporting its potential for further translational and clinical evaluation in DCM (jRCT 2033240447).

## Electronic Supplementary Material

Below is the link to the electronic supplementary material.

Supplementary Material 1.

Supplementary Material 2.

Supplementary Material 3.

Supplementary Material 4.

Supplementary Material 5.

Supplementary Material 6.

Supplementary Material 7.

Supplementary Material 8.

Supplementary Material 9.

Supplementary Material 10.

Supplementary Material 11.

Supplementary Material 12.

## Data Availability

The datasets used and/or analyzed during the current study are available from the corresponding author on reasonable request.

## Abbreviations

CO: Cardiac output
DCM: Dilated cardiomyopathy
FS: Fraction shortening
HE: Hematoxylin-Eosin
HR: Heart rate
ICM: Ischemic cardiomyopathy
INT: IHJ-301 implantation or sham operation
iPSC: Induced pluripotent stem cell
jRCT: Japan Registry of Clinical Trials
LVAD: Left ventricular assist device
LVEF: Left ventricular ejection fraction
LVIDd: Left ventricular end-diastolic diameter
LVIDs: Left ventricular end-systolic diameters
MT: Masson-trichrome
PAP: Pulmonary artery pressure
PCWP: Pulmonary artery wedge pressure
RAP: Right atrial pressure
SV: Stroke volume
SDPHF: Step-Down Pacing Heart Failure
TAC: Tacrolimus

## Declarations

### Generative AI and AI-assisted technologies in the writing process

AI-assisted tools were used solely for language editing to improve clarity and grammar. The authors take full responsibility for the content of the manuscript, including the accuracy of the data and the integrity of the conclusions.
