# Supplementary material for: Efficacy of multi-layered human iPS cell-derived cardiovascular cell sheets in a pacing-induced canine dilated cardiomyopathy model
Source: Stem Cell Res Ther. 2026 Aug 19;17:263. doi: 10.1186/s13287-026-05207-x (PMC13488229; doi:10.1186/s13287-026-05207-x)
Supplement: Supplementary file 1 [file 13287_2026_5207_MOESM8_ESM.pdf]

ARRIVE 2.0 Checklist

Study title: Efficacy of IHJ-301 implanted to the left ventricle in a canine pacing-induced heart failure model

1. Study design

Two parallel groups were compared: Control (pericardiotomy + physiological tissue adhesive spray only) and Test (IHJ-301 implanted to the epicardial surface of the left ventricle). Experimental unit: one dog. A pacemaker-induced chronic tachyarrhythmia heart failure model was established prior to allocation.

2. Sample size

n=6 dogs per group at allocation (total n=12).
One dog in the Test group was euthanized at Day 14 after IHJ-301 implantation due to gastrointestinal adverse events judged related to immunosuppression, so analyses used Control n=6 and Test n=5.
A priori sample size calculation was performed using the change in ejection fraction (ΔEF) as the primary endpoint.
Effect size (mean difference = 13.9 ± 3.9 %) was estimated from our previous studies (doi:10.1371/journal.pone.0201650.) and the results of other studies (doi:10.1016/j.jtcvs.2006.01.024., doi:10.1253/circj.CJ-12-0615.). Using $\alpha=0.05$ (two-sided) and power=0.8, considering a 50% reduction in expected effect size to account for biological variability and potential dropouts, a minimum of n=6 animals per group was required. The calculation was performed using R Commander (version 2.7-1).

3. Inclusion and exclusion criteria

Inclusion (pre-specified): Beagle dogs with reduced left ventricular ejection fraction (EF 32.3–49.7%) 27–28 days after pacemaker activation, and clinically normal during quarantine/acclimation.
Exclusion: No animals were excluded at allocation. One Test animal reached a humane endpoint due to persistent anorexia, diarrhea, and melena (necropsy findings were consistent with a gastrointestinal etiology) and was therefore excluded from the analysis.
Exact numbers included in each analysis were: Control n = 6, Test n = 5.

4. Randomization

Allocation was performed at grouping (Day 27/28 after pacing) using a deterministic minimization approach to balance baseline echocardiographic indices (EF and LVIDd) between the two arms. Because only four animals could undergo surgery per day, block allocation was applied (block size = 4; Control n = 2, Test n = 2), and animals were assigned such that the between-group differences in daily mean EF and LVIDd were

minimized. Although a formal computer-generated random sequence or stochastic element was not used, this structured allocation ensured comparable baseline cardiac function across groups. Allocation concealment was not feasible due to the operational constraints of daily surgical scheduling.

Potential confounders, including cage location and measurement order, were controlled by rotating cages and implementing balanced measurement schedules throughout the study.

5. Blinding

Because the number of animals that could undergo surgery per day was limited, the surgical schedule and observation dates had to be fixed in advance. Consequently, staff necessarily knew which animals were operated on each day, making full blinding operationally impracticable. In addition, immunosuppressants required weight-based dose adjustment for each animal, and treatment regimens differed between groups; therefore, treatment administration could not be performed under blinded conditions.

To mitigate potential bias, we implemented partial blinding by assigning independent personnel to different stages of the study: the surgeon (procedures), the measurer (instrumental measurements), the outcome assessor (data evaluation), and the animal caretakers (husbandry). Group allocation and treatment details were not shared across roles, and each person performed their tasks without access to information beyond what was operationally required.

Data analysis was conducted by an independent statistician who was not involved in surgery or animal care, using datasets in which group identifiers had been coded.

6. Outcome measures

Primary outcomes: change in EF (ΔEF), change in cardiac output (ΔCO), and change in stroke volume (ΔSV) from the day of IHJ-301 implantation (before implantation) to 4 weeks after implantation. Secondary outcomes: LVIDd/LVIDs/%FS, RAP/PAP/PCWP/HR, serial blood biochemistry (ASAT, ALAT, ALP, LDH, LAP, ChE, γ-GTP, TB, TP, Alb, A/G, TC, TG, BUN, UA, Cre), organ and lung weights/ratios, LV fibrosis area (%), and histopathological findings.

7. Statistical methods

Group data are presented as mean ± SE. Variance equality was assessed using the F-test. For variables that satisfied the assumptions of normality and homoscedasticity, comparisons were performed using the Student's t-test or paired t-test. For variables that did not meet these assumptions, the primary comparison was conducted using the Mann–Whitney U test. All analyses were performed using R Commander (version 2.7-1).

8. Experimental animals

Species/strain/sex: Dog (Beagle), male; body weight 9.85–11.75 kg at pacemaker implantation. Source: Kitayama Labes Co., Ltd. Health status: conventional; quarantine and veterinary checks conducted. Animal IDs and per-animal data provided. Total animals: 12.

9. Experimental procedures

Heart failure induction: subcutaneously implanted pacemaker (SIP-501) activated 226–237 bpm for ~4 weeks, then 206–217 bpm post-affixation. Surgery: under general anesthesia (thiopental induction; isoflurane maintenance), left thoracotomy, pericardiotomy; IHJ-301 affixed on LV with physiological tissue adhesive; chest closed; postoperative antibiotics and analgesia per protocol. Immunosuppression: tacrolimus 1 mg/kg/day, mycophenolate mofetil 20 mg/kg/day, prednisolone 1 mg/kg/day from 3–4 days pre-affixation to necropsy (with forced dosing for one animal). Monitoring: daily clinical observation; scheduled body weights; echocardiography (Vivid S6) at pre-pacing, Day 27/28 (grouping), and 2 & 4 weeks post-affixation; hemodynamics (RAP, PAP, PCWP, CO, HR, SV) pre-affixation and 4 weeks after affixation (LabChart Pro); serial blood draws pre- and weekly to 4 weeks; necropsy at 4 weeks (organ weights, LV fibrosis area by Masson-trichrome using Win ROOF 2013).

10. Results

At 4 weeks post-implantation, ΔEF was significantly higher in Test than Control (10.4 ± 1.5% vs 1.9 ± 0.3%, $p<0.01$). ΔCO and ΔSV also favored Test (ΔCO 0.22 ± 0.19 vs −0.58 ± 0.12 L/min, $p=0.007$; ΔSV 1.25 ± 1.26 vs −2.99 ± 0.60 mL/beat, $p=0.011$). EF improved within Test group from post-pacing to 4 weeks ($p=0.017$). No significant differences in organ weights or LV fibrosis area (Control 1.0 ± 0.2% vs Test 0.6 ± 0.1%). Blood biochemistry differences were observed at some timepoints (e.g., TP, Alb, TC, LAP, BUN) but remained within reference expectations and were judged clinically non-meaningful in the report. One Test animal met humane endpoint due to GI symptoms likely related to immunosuppression; no heart-failure related mortality occurred.

Ethics and approvals: All procedures were conducted in accordance with institutional and national guidelines. Animal experiments were approved by the institutional animal care and use committee (Approval No. 2023-183). Genetic modification procedures for animal experiments were approved by the institutional biosafety committee (Approval No. 2024FEB09). The use of human induced pluripotent stem cells was approved by the Center for iPS Cell Research and Application (CiRA), Kyoto University (Approval No. G1025-8).

Housing & husbandry: Individual stainless cages; room 1-107; 17.5–28.4°C; 48.6–89.4% humidity; 10–15 air changes/h; 12-h light (07:00–19:00); DS-A diet (Orienta), municipal water with automatic supply; enrichment with natural rubber toys (KONG).

Software & instruments: Echocardiography (Vivid S6, GE; 11 MHz sector probe); hemodynamics (RMT-1000; LabChart Pro, AD Instruments); pacemaker SIP-501; anesthesia systems NS-5000A; monitors BSM-3592.
