# Supplementary material for: Efficacy of multi-layered human iPS cell-derived cardiovascular cell sheets in a pacing-induced canine dilated cardiomyopathy model
Source: Stem Cell Res Ther. 2026 Aug 19;17:263. doi: 10.1186/s13287-026-05207-x (PMC13488229; doi:10.1186/s13287-026-05207-x)
Supplement: Supplementary file 2 [file 13287_2026_5207_MOESM12_ESM.docx]

**Additional information**

Titles and legends for Additional files: Videos S1-6

Additional file 1: Video S1　 **Echocardiographic video 1**

Echocardiographic video of sham operated animal shown in Fig. 2b as black triangle at Pre-pacing. Pre-pacing: the day of pacemaker implantation (before pacemaker implantation).

Additional file 2: Video S2　 **Echocardiographic video 2**

Echocardiographic video of sham operated animal shown in Fig. 2b as black triangle at 0W. 0W: the day of INT (before INT),

Additional file 3: Video S3　 **Echocardiographic vide 3**

Echocardiographic video of sham operated animal shown in Fig. 2b as black triangle at 4W. 4W: 4 weeks after INT.

Additional file 4: Video S4　 **Echocardiographic video 4**

Echocardiographic video of IHJ-301 implanted animal shown in Fig. 2b as red triangle at Pre-pacing. Pre-pacing: the day of pacemaker implantation (before pacemaker implantation).

Additional file 5: Video S5　 **Echocardiographic video 5**

Echocardiographic video of IHJ-301 implanted animal shown in Fig. 2b as red triangle at 0W. 0W: the day of INT (before implantation),

Additional file 6: Video S6　 **Echocardiographic video 6**

Echocardiographic video of IHJ-301 implanted animal shown in Fig. 2b as red triangle at 4W. 4W: 4 weeks after INT.
